# CCND2 mRNA Expression Is Correlated With R-CHOP Treatment Efficacy and Prognosis in Patients With ABC-DLBCL

**DOI:** 10.3389/fonc.2020.01180

**Published:** 2020-07-21

**Authors:** Di Wang, Yue Zhang, Yi-Qun Che

**Affiliations:** ^1^Department of Clinical Laboratory, National Cancer Center/National Clinical Research Center for Cancer/Cancer Hospital, Chinese Academy of Medical Sciences and Peking Union Medical College, Beijing, China; ^2^State Key Laboratory of Molecular Oncology, National Cancer Center/National Clinical Research Center for Cancer/Cancer Hospital, Chinese Academy of Medical Sciences and Peking Union Medical College, Beijing, China

**Keywords:** ABC-DLBCL, RNA *in situ* hybridization, overall survival, CCND2, progression-free survival

## Abstract

In this study we investigated whether the expression of cyclin D2 (CCND2) mRNA in activated B-cell-like diffuse large B-cell lymphoma (ABC-DLBCL) was correlated with the efficacy of Rituximab combined with chemotherapy (R-CHOP) treatment and patient prognosis. Tissue microarray and RNAscope *in situ* hybridization were used to detect CCND2 mRNA expression in 117 ABC-DLBCL tumor tissues and associations between CCND2 expression and progression-free survival was analyzed. We also downloaded data from the Gene Expression Omnibus database to analyze CCND2 expression and the efficacy of R-CHOP treatment and prognosis of patients with newly diagnosed ABC-DLBCL. The positive expression rate of CCND2 mRNA in patients with ABC-DLBCL was 41%. Progression-free survival was significantly lower in patients with positive rather than those negative CCND2 expression (*P* = 0.005). Further, R-CHOP treatment was significantly more effective for patients with ABC-DLBCL with high CCND2 mRNA expression than those with low expression (*P* = 0.039). Multivariate regression analysis suggested that high CCND2 expression was an independent prognostic risk factor for progression-free survival for patients with ABC-DLBCL who achieved complete remission after R-CHOP treatment. CCND2 expression in ABC-DLBCL tumors, detected by RNA *in situ* hybridization, is closely related to the curative effect of R-CHOP and patient prognosis following R-CHOP treatment, and represents a potential biomarker for treatment efficacy and prognostic evaluation in patients with ABC-DLBCL.

## Introduction

Non-Hodgkin's lymphoma (NHL) is a common malignant tumor of lymphoid tissues, responsible for morbidity and mortality, ranking seventh and ninth, respectively, among malignant tumors in western developed countries (1). In recent years, the incidence of NHL has also increased significantly in China (2). Among NHL subtypes, diffuse large B-cell lymphoma (DLBCL) is the most common, and can be divided into activated B-cell-like (ABC)-DLBCL, germinal center B-cell-like (GCB)-DLBCL and unclassified according to its different cellular origins; ABC-DLBCL is associated with poorer prognosis (3). The proportion of NHL accounted for by ABC-DLBCL among Chinese patients, is much higher than that in individuals from European and American countries. Currently, the accepted first-line treatment regimen for DLBCL is rituximab combined with chemotherapy (R-CHOP). The R-CHOP regimen can significantly improve the outcome for patients with DLBCL; however, the overall cure rate is only ~60%, and long-term progression-free survival of patients with ABC-DLBCL following first-line treatment remains <50% (4). The latest edition of the National Comprehensive Cancer Network guidelines also clearly state that tissue biopsies must be collected from newly diagnosed patients to determine the tumor's cellular origin (5). Therefore, for patients with ABC-DLBCL, screening of tumor markers related to the efficacy of R-CHOP treatment, timely prediction of outcome, and exploration of available second-line treatment options are important clinical issues that need to be addressed urgently.

Transcriptome sequencing and bioinformatics analysis demonstrated that CCND2 mRNA expression differed significantly in ABC-DLBCL specimens from patients treated with R-CHOP according to differing prognosis in the early stage of our research group. Cyclin D2 (CCND2) is a D-type cyclin with key roles in cell cycle regulation, differentiation, and malignant transformation (6). CCND2 is abnormally expressed in a variety of malignant tumors, including colorectal, prostate, and bladder cancers (7–9). The function of CCND2 is primarily related to the regulation of cell cycle, however, it is unclear whether CCND2 expression can influence the efficacy of R-CHOP treatment and patient prognosis in DLBCL. In this study, we used tissue microarray and RNA *in situ* hybridization to detect the expression of CCND2 mRNA in ABC-DLBCL tissue specimens and analyzed the correlation between CCND2 levels and prognosis in patients with ABC-DLBCL. In addition, we explored whether CCND2 mRNA expression can be used as a molecular marker for the prognosis of patients with ABC-DLBCL treated with R-CHOP.

## Materials and Methods

### Patients and Clinical Data

Tumor samples were collected from 117 patients with newly diagnosed ABC-DLBCL treated at the National Cancer Center/Cancer Hospital, Chinese Academy of Medical Sciences (Beijing, China). Enrolment criteria were as follows: (1) ABC-DLBCL diagnosed by pathology, ABC status was determined by immunohistochemical (IHC) stain algorithm and primary mediastinal large B-cell lymphoma, testicular, primary DLBCL of the central nervous system were excluded; (2) 6–8 cycles of R-CHOP treatment; (3) complete clinical prognosis data; (4) no heart, liver, renal, digestive, or metabolic disorders. This study was approved by the Ethics Committee of the Cancer Hospital of the Chinese Academy of Medical Sciences. Written informed consent was provided by all patients prior to enrollment in the study.

### Tissue Microarray

Collected tissues stored at −80°C were fixed in formalin before paraffin embedding and cutting into 4 μm thick sections, staining with hematoxylin and eosin, and marking of the tumor area under a microscope. Tumor areas were prepared as wax blocks and re-embedded. Two tissue samples were taken from each tumor, and the wax blocks cut into 5 μm tissue sheets for use in microarray analysis.

### Detection of CCND2 mRNA by RNAscope *In situ* Hybridization

RNAscope *in situ* hybridization was used to analyze the prepared tissue chips. The human CCND2 gene probe (ACD #470031) was purchased from Advanced Cell Diagnostics (Newark, LA). Tissue chips were dewaxed with xylene, hydrated in an ethanol gradient, endogenous peroxidase activity blocked with hydrogen peroxide for 10 min, and target retrieval solution applied for 15 min. Sections were then subjected to RNAscope probe hybridization and amplification; DAB was used for color development, and hematoxylin for counterstaining, followed by ethanol gradient dehydration, clearing using xylene, and mounting. Specific processes were performed according to the instructions for the RNAscope 2.5 HD Reagent Kit-BROWN (ACD #322300). A NanoZoomer pathology scanner (Hamamatsu Corporation, Japan) was used for high-precision scanning of the tissue microarray, and blind evaluation performed by two pathologists. mRNA signal scores were determined semi-quantitatively; that is, as a combination of signal intensity (0, negative; 1, weak positive; 2, medium positive; 3, strong positive) and the proportion of positive cells (0, 0%; 1, 1–20%; 2, 21–50%; 3, 51–100%), with the product of the two scores the final score for the specimen. A final score of >3 was considered positive for CCND2 mRNA expression.

### Microarray Data Preprocessing

Raw microarray data files (.CEL files) and corresponding clinical data from the GSE10846 and GSE31312 datasets were retrieved from the Gene Expression Omnibus (GEO) database (platform GPL570). Raw microarray data from gene chips were normalized using the MAS5.0 algorithm by the “affy” package. The “SVA” package (in R software) was used to control for batch effects. After excluded those without clinical information, a total of 884 DLBCL patients were enrolled in our study (details in Table 1).

**Table 1:** Basic clinical information of data in Gene Expression Omnibus.

**Features**	**Group**	**GSE10846 (*n* = 414)**	**GSE31312 (*n* = 470)**
Age	≤60	188	200
	>60	226	270
Gender	Female	172	199
	Male	224	271
	NA	18	/
Subtype	GCB	183	227
	ABC	167	199
	UC	64	44
Stage	I–II	188	220
	III–IV	218	250
	NA	8	/
ECOG	≤1	296	374
	>1	93	96
	NA	25	/
LDH	Normal	173	148
	Elevated	178	278
	NA	63	44
Extranodal involvement	≤1	353	366
	>1	30	104
	NA	31	/
Treatment	R-CHOP	233	470
	CHOP	181	/
Response	CR	/	354
	No-CR	/	116
B-symptoms	No	/	276
	Yes	/	132
	NA	/	62
IPI score	<2	132	137
	≥2	189	289
	NA	93	44

*ECOG, Eastern Cooperative Oncology Group performance status; LDH, lactate dehydrogenase; IPI, International Prognostic Index*.

### Data Analysis

Statistical analyses were performed using SPSS 22.0 software. The Mann-Whitney *U*-test and *T*-test were used to evaluate the significance of differences between two groups. The χ^2^-test were used to analyze relationships between gene expression and clinical parameters. Survival curves were constructed using the Kaplan-Meier method and compared using the log-rank test. The Cox proportional hazards model was applied for univariate and multivariate analyses. Candidate prognostic factors, with a significance level ≤0.10 in univariate analysis, were included in the multivariate analysis. Risk is expressed as hazard ratio (HR) and 95% confidence interval (CI). All statistical tests were two-sided, and considered significant when *P*-values were < 0.05.

## Results

### Relationship Between CCND2 mRNA Expression and Clinicopathological Factors in Patients With ABC-DLBCL

**Figure 1:**
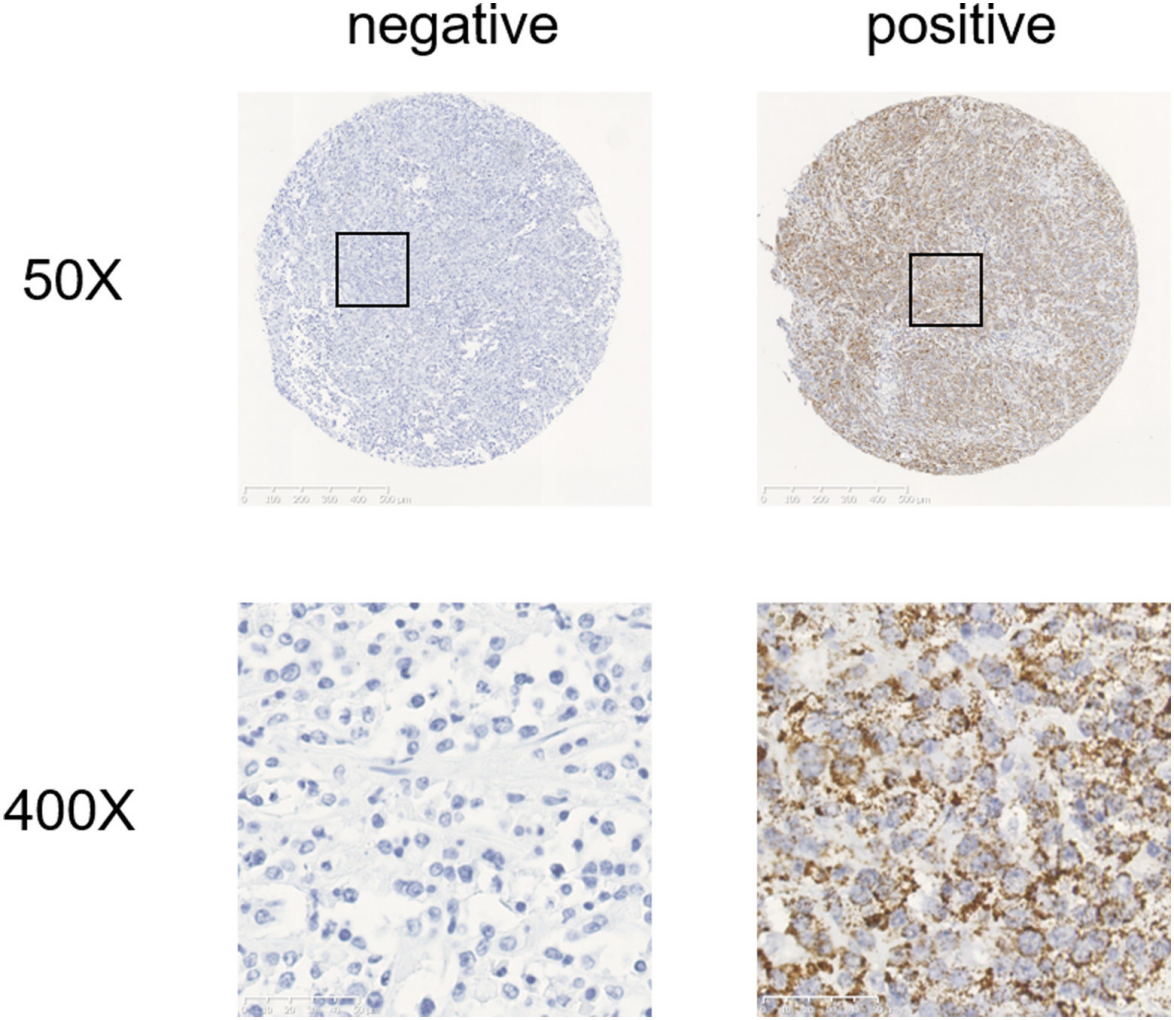
Representative RNA *in situ* hybridization images of samples negative and positive for CCND2 mRNA expression.

We performed RNAscope *in situ* hybridization on paraffin tissue samples from 117 patients newly diagnosed with ABC-DLBCL who received R-CHOP treatment. The positive rate for CCND2 mRNA expression was 41% (48/117) (Figure 1). There were significant differences in clinical stage (*P* = 0.003) and extra-nodal involvement (*P* = 0.024) between patients with ABC-DLBCL negative and positive for CCND2 mRNA (Table 2).

**Table 2:** Association between CCND2 expression level and clinical characteristics.

**Clinical characteristic**		**CCND2**		***P***
		**Negative**	**Positive**	
Gender	Male	36	28	0.510
	Female	33	20	
Age (years)	≤60	49	33	0.792
	>60	20	15	
Clinical stage	I–II	43	19	**0.003**
	III–IV	19	28	
ECOG	<2	46	40	1.000^*^
	≥2	3	3	
Extranodal involvement	≤1	40	26	**0.024**
	>1	9	17	
LDH	Normal	32	23	0.423
	Elevated	21	21	
IPI score	<2	35	23	0.133
	≥2	17	21	

* *Fisher's Exact Test*.

*ECOG, Eastern Cooperative Oncology Group performance status; LDH, lactate dehydrogenase; IPI, International Prognostic Index. Bold indicate P value is less than 0.05*.

### Relationship Between CCND2 mRNA Expression and Progression-Free Survival in Patients With ABC-DLBCL

**Figure 2:**
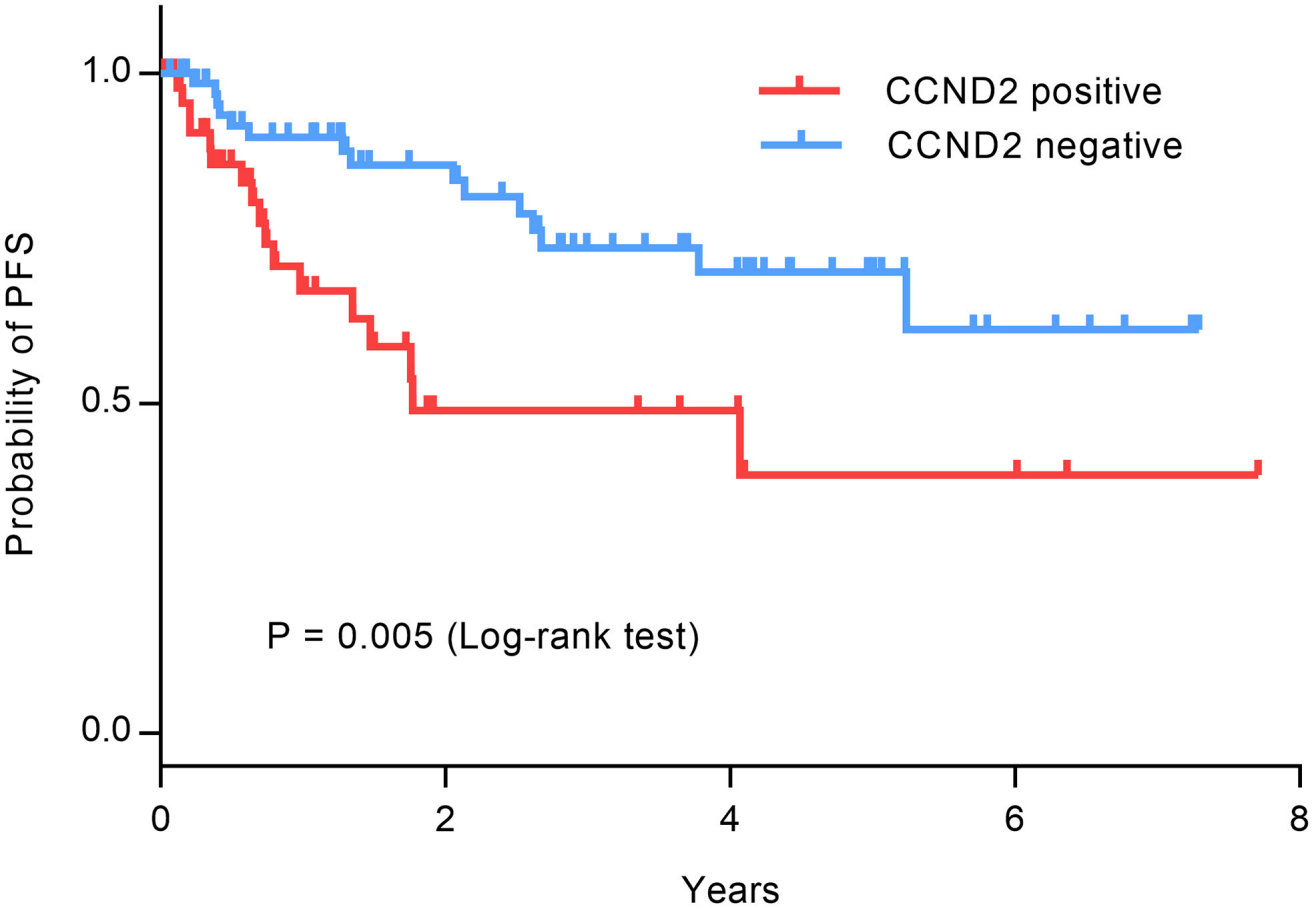
Survival curves of patients with ABC-DLBCL positive (*n* = 47; red line) and negative (*n* = 68; blue line) for CCND2 mRNA expression.

Patients newly diagnosed with ABC-DLBCL undergo standard R-CHOP as the first-line treatment, and follow-up evaluations are performed regularly every 3 months by CT to determine whether recurrence or progression occurred. All patients were followed until recurrence or the end of this study on December 30, 2018. Analysis of follow-up data demonstrated that patients with ABC-DLBCL negative for CCND2 expression had significantly superior progression-free survival than those positive for CCND2 mRNA (χ^2^ = 8.005, *P* = 0.005; Figure 2).

### Correlation Between CCND2 mRNA Expression and R-CHOP Efficacy in Patients With ABC-DLBCL

Next, we analyzed data downloaded from the GEO database to evaluate the relationship between CCND2 mRNA expression and clinical and pathological parameters. We found that, among patients with ABC-DLBCL treated using R-CHOP chemotherapy, relative CCND2 mRNA expression levels were significantly higher in those exhibiting curative control (i.e., complete remission) relative to those who did not achieve complete remission (*P* = 0.039; Table 3).

**Table 3:** Association between CCND2 expression level and clinical characteristics from GEO data.

**Characteristic**	**Group**	**CCND2 expression in GCB-DLBCL**	***P***	**CCND2 expression in ABC-DLBCL**	***P***
Age (years)	≤60	8.70 (1.13)	0.781	9.61 ± 1.21	0.176^*^
	>60	8.71 (1.19)		9.79 ± 1.19	
Gender	Female	8.70 (1.25)	0.589	9.68 ± 1.08	0.470^*^
	Male	8.71 (1.09)		9.77 ± 1.29	
Stage	I–II	8.62 (1.10)	0.054	9.79 ± 1.19	0.376^*^
	III–IV	8.87 (1.19)		9.67 ± 1.21	
Extranodal involvement	≤1	8.71 (1.16)	0.688	9.74 ± 1.19	0.711^*^
	>1	8.53 (1.22)		9.67 ± 1.22	
ECOG	≤1	8.75 (1.15)	0.056	9.67 ± 1.14	0.311
	>1	8.41 (1.28)		9.82 ± 1.35	
LDH	Normal	8.85 (1.15)	**0.009**	9.78 ± 1.11	0.278^*^
	Elevated	8.51 (1.20)		9.63 ± 1.22	
B-symptoms	No	8.72 ± 0.86	0.594^*^	9.73 ± 1.13	0.809^*^
	Yes	8.79 ± 0.79		9.77 ± 1.18	
IPI score	<2	8.71 (1.10)	0.252	9.84 ± 1.07	0.155^*^
	≥2	8.60 (1.23)		9.63 ± 1.21	
R-CHOP efficacy evaluation	CR	8.75 (1.08)	0.245	9.80 ± 1.10	0.039^*^
	No-CR	8.64 (1.53)		9.40 ± 1.30	

* *T-test*.

*GCB-DLBCL, Germinal center B-cell-like diffuse large B-cell lymphoma; ABC-DLBCL, activated B-cell-like diffuse large B-cell lymphoma; ECOG, Eastern Cooperative Oncology Group performance status; LDH, lactate dehydrogenase; CR, complete remission; IPI, International Prognostic Index. Bold indicate P value is less than 0.05*.

### High CCND2 mRNA Expression Is an Independent Prognostic Factor for ABC-DLBCL Patients Who Achieve Complete Remission After R-CHOP Treatment

To assess whether CCND2 is an independent prognostic factor for ABC-DLBCL patients who achieved complete remission using the R-CHOP regimen, univariate and multivariate Cox regression analysis was performed. The results demonstrated that IPI score (HR, 5.871; 95% CI, 2.278–15.131; *P* = 0.0002) was an independent prognostic factor for duration of overall survival. And CCND2 mRNA expression has a trend (HR, 1.311; 95% CI, 0.992–1.733; *P* = 0.057; Table 4). Further, IPI score (HR, 2.867; 95% CI, 1.432–5.742; *P* = 0.003) and CCND2 expression (HR, 1.390; 95% CI, 1.093–1.767; *P* = 0.007) were also independent prognostic factors for progression-free survival (Table 5).

**Table 4:** Univariate and multivariate analysis to identify factors prognostic for overall survival.

**Characteristic**	**Univariate cox**			**Multivariate cox**		
	**HR**	**95% CI**	***P***	**HR**	**95% CI**	***P***
CCND2 expression	1.308	1.007–1.700	**0.044**	1.311	0.992–1.733	0.057
Gender (female vs. male)	0.640	0.359–1.143	0.132			
IPI score (≥2 vs. <2)	6.143	2.373–15.904	**0.0002**	5.871	2.278–15.131	**0.0002**
B-symptoms (no vs. yes)	1.382	0.716–2.665	0.335			

*ECOG, Eastern Cooperative Oncology Group performance status; LDH, lactate dehydrogenase; HR, hazard ratio; CI, confidence interval; IPI, International Prognostic Index. Bold indicate P value is less than 0.05*.

**Table 5:** Univariate and multivariate analysis for factors prognostic for progression-free survival.

**Characteristic**	**Univariate cox**			**Multivariate cox**		
	**HR**	**95% CI**	***P***	**HR**	**95% CI**	***P***
CCND2 expression	1.317	1.042–1.664	**0.021**	1.390	1.093–1.767	**0.007**
Gender (female vs. male)	0.705	0.416–1.195	0.194			
IPI score (≥2 vs. <2)	2.870	1.435–5.741	**0.003**	2.867	1.432–5.742	**0.003**
B-symptoms (no vs. yes)	1.039	0.563–1.920	0.902			

*ECOG, Eastern Cooperative Oncology Group performance status; LDH, lactate dehydrogenase; HR, hazard ratio; CI, confidence interval; IPI, International Prognostic Index. Bold indicate P value is less than 0.05*.

## Discussion

Diffuse large B-cell lymphoma describes a very heterogeneous group of tumors. The DLBCL subtypes are established as differing in terms of chromosome alterations, signaling pathway activation, and clinical outcomes. GCB-DLBCL is characterized by persistent somatic hypermutation, BCL6 up-regulation, and almost universal CD10 expression, while ABC-DLBCL is associated with chronic activated B cell receptor signaling and NF-κB dysregulation (10). The R-CHOP regimen, as the standard first-line treatment regimen for DLBCL, is widely used clinically; however, treatment efficacy and prognosis differ significantly among patients. About 20–50% of DLBCL patients will be refractory to R-CHOP or will relapse after achieving complete response (CR) (11). The prognosis of relapsed/refractory patients with ineffective second-line therapy or intolerance to transplantation is extremely poor, all of which have led to a continuous increase in the mortality of DLBCL (12, 13). Consequently, factors influencing the response of R-CHOP treatment and predict relapse and refractory of patients are the subject of increasing research.

In this study, we initially collected tumor tissue samples from patients with poor and good prognosis by microdissection. We conducted analysis using a multiple nucleic acid *in situ* hybridization technology-RNAscope technology, which can provide high specificity and sensitivity to determine the spatial localization of target mRNA in a single cell, and combined with tissue morphology can generate accurate information regarding RNA expression by different types of cells in the tissue (14). RNAscope technology can detect target RNA molecules in paraffin-embedded tissues, using double “Z” probes (the target RNA recognition sequence is ~18–25 bases long) and a signal amplification system to ensure the specificity and strength of the signal. In addition, this technique can compensate for the effect of partial RNA degradation in fixed and embedded samples on signal intensity using multiple short probes to cover longer fragments of the target RNA (15). Therefore, the technology can obtain accurate results from paraffin sections, which are easy to generate and widely available clinically, greatly increasing the popularity of this technology in the clinic. Increasing numbers of studies are using RNAscope *in situ* hybridization technology to identify mRNA molecules related to prognosis in patients with cancer (16, 17).

CCND2 (Cyclin D2), is a member of the Cyclin family, along with Cyclin D1, Cyclin D3, and Cyclin E, which act as cell cycle regulatory proteins. CCND2 promotes cell cycle progression by binding and activating cyclin-dependent kinase 4 (cdk4)/cdk6. The activated CCND2-cdk4/cdk6 complex over-phosphorylates the tumor suppressor protein pRB, promotes the release, and activation of the transcription factor E2F, and targets the production of several proteins required to regulate cell cycle progression (18). Therefore, CCND2 is a key regulator involved in accelerating the cell cycle, and its abnormal expression may cause dysregulated cell proliferation (19, 20). Alizadeh et al. used microarray chips to analyze differences in gene expression between DLBCL and normal human lymphocyte subsets under a series of activation conditions. They found that CCND2 is expressed at high levels in DLBCL (21). Then some studies have appeared to detect the expression of CCND2 in DLBCL by immunohistochemical staining, and the positive rate is low. Hans et al. found that the positive rate of CCND2 expression in non-GCB patients was 14%, and the positive expression of CCND2 was associated with poor OS and PFS (22). Amen et al. also analyzed the positive rate of CCND2 expression in DLBCL patients by immunohistochemistry. The positive rate was 24.7%, and it was related to the prognosis of patients (23). In this study, we constructed tissue microarrays by collecting paraffin-embedded tissue specimens from 117 patients with ABC-DLBCL, used RNAscope technology to detect CCND2 mRNA expression *in situ*. Our results showed that the positive rate for CCND2 mRNA expression was 41% and the high expression of CCND2 mRNA was negatively correlated with prognosis in ABC-DLBCL patients receiving R-CHOP (*P* = 0.005). The difference in the positive rate may be due to the different performance of the protein antibodies. The high sensitivity of the RNAscope technology used in our study can more accurately detect the expression of CCND2 mRNA in tumor tissue *in situ*. In addition, ABC-DLBCL expresses high levels of downstream target genes of NF-κB, including IκBα and cyclin D2 (24). *In vitro* experiments show that NF-κB activity is necessary for cell cycle function. The CCND2 gene promoter contains two NF-κB binding sites and inhibition of NF-κB signaling causes G1 phase arrest of ABC-DLBCL (25). As cell cycle arrest causes apoptosis in many tumor types, targeting CCND2 expression may contribute to the cytotoxic effects of R-CHOP. Therefore, molecular targeted therapies that inhibit the components of the NF-κB/cyclin D2 pathway can be a potential treatment option for ABC-DLBCL patients with poor prognosis.

There are very few researches on the correlation between the expression of CCND2 and the therapeutic effect of R-CHOP and the prognostic significance of ABC-DLBCL patients who achieve CR after R-CHOP treatment. To explore this issue, we downloaded two data sets (GSE10846 and GSE31312) from the GEO database to study the correlation between CCND2 mRNA expression and the efficacy and prognosis of R-CHOP treatment. Interestingly, database results display that patients with high expression of CCND2 mRNA after R-CHOP standard treatment have better efficacy than those with low expression (*P* = 0.039). We speculate that this result may be due to use of the chemotherapy drugs, vincristine, and cyclophosphamide, which target the cell cycle in the R-CHOP regimen. These chemotherapeutic drugs may respond more strongly to tumor tissues with high CCND2 mRNA expression, making them more effective for patients with such tumors. We will design experiments to verify this hypothesis in the future. In addition, the multivariate regression analysis of GEO data also suggested that in patients with ABC-DLBCL who achieved complete remission after R-CHOP treatment, high CCND2 expression was an independent progression-free survival risk factor. Garcia et al. (26) studied anomalous extracellular mRNAs in plasma from DLBCL patients with R-CHOP treatment. In plasma from patients with complete response to treatment, they illustrated that patients with presence of CCND2 mRNA had shorter PFS than patients without the marker. Although the CCND2 mRNA was detected in plasma in this study, the results are consistent with ours.

In summary, we found that high CCND2 mRNA expression was negatively correlated with prognosis in patients with ABC-DLBCL receiving R-CHOP. Further, we demonstrate that CCND2 level is an independent prognostic risk factor for progression-free survival of patients with ABC-DLBCL following R-CHOP therapy and achieved complete remission. In future, we will conduct further study of the efficacy of targeted molecular therapy that inhibits CCND2 expression combined with R-CHOP in patients with ABC-DLBCL, with a view to further improving the prognosis of patients with this disease.

## Data Availability Statement

The raw data supporting the conclusions of this article will be made available by the authors, without undue reservation.

## Ethics Statement

The studies involving human participants were reviewed and approved by Cancer Hospital of the Chinese Academy of Medical Sciences. The patients/participants provided their written informed consent to participate in this study.

## Author Contributions

Y-QC: conception and design and administrative support. DW: provision of study materials or patients. YZ: data analysis and interpretation. Y-QC and DW: manuscript writing. All authors: final approval of manuscript.

## Conflict of Interest

The authors declare that the research was conducted in the absence of any commercial or financial relationships that could be construed as a potential conflict of interest.
